# Lessons from the analysis of nonhuman primates for understanding human aging and neurodegenerative diseases

**DOI:** 10.3389/fnins.2015.00064

**Published:** 2015-03-04

**Authors:** Jean-Michel Verdier, Isabelle Acquatella, Corinne Lautier, Gina Devau, Stéphanie Trouche, Christelle Lasbleiz, Nadine Mestre-Francés

**Affiliations:** ^1^Université de MontpellierMontpellier, France; ^2^Institut National de la Santé et de la Recherche Médicale, U1198Montpellier, France; ^3^Ecole Pratique des Hautes EtudesParis, France

**Keywords:** neuroscience, brain, aging, neurodegenerative diseases, nonhuman primate

## Abstract

Animal models are necessary tools for solving the most serious challenges facing medical research. In aging and neurodegenerative disease studies, rodents occupy a place of choice. However, the most challenging questions about longevity, the complexity and functioning of brain networks or social intelligence can almost only be investigated in nonhuman primates. Beside the fact that their brain structure is much closer to that of humans, they develop highly complex cognitive strategies and they are visually-oriented like humans. For these reasons, they deserve consideration, although their management and care are more complicated and the related costs much higher. Despite these caveats, considerable scientific advances have been possible using nonhuman primates. This review concisely summarizes their role in the study of aging and of the mechanisms involved in neurodegenerative disorders associated mainly with cognitive dysfunctions (Alzheimer's and prion diseases) or motor deficits (Parkinson's and related diseases).

## Why do we need animal models?

The simplest answer to this question is to increase our general knowledge, to experimentally test theories. Animal model usefulness is manifold, from the study of physiological processes to the identification of disease-causing mechanisms. Indeed, physiopathological studies are of the utmost importance for developing diagnostic and therapeutic approaches based on the discovery of new, more sensitive and specific biomarkers, the identification of the mechanism of action of drugs, the establishment of pharmacodynamics and pharmacokinetic parameters, the toxicity analysis of new compounds or the assessment of clinical drug regimens.

Many different animal models, ranging from unicellular organisms (bacteria, yeast) to invertebrates (the roundworm *Caenorhabditis elegans* or the fruit fly *Drosophila melanogaster*) and vertebrates (fish and mammals), are currently used for research on aging and neurodegenerative disorders. They are all of interest and importance, but they also show limitations and drawbacks. Most studies on neurodegenerative diseases have been done in transgenic animals (Gama Sosa et al., 2012), particularly in mice. Indeed, as the production and handling of transgenic mice is currently quite easy, they have played and continue to play a very important role in biomedical research. Nevertheless, fundamental differences between rodents and humans exist. Conversely, nonhuman primates (NHPs) share many structural and functional features with humans. NHPs diverged from humans and formed various lineages: Great Apes (our closest relatives, *Hominidae—*e.g., chimpanzees), Old World monkeys (*Cercopithecidae—*e.g., baboons, macaques), New World monkeys (*Parvorder Platyrrhini—*e.g., marmosets) and Prosimians (our most distant relatives, the Suborder *Strepsirrhini* and Infraorder *Tarsiiformes—*e.g., lemurs). Therefore, NHPs are genetically the closest species to humans (Kumar and Hedges, 1998; Finch and Austad, 2012). The purpose of this review is to explain why the use of NHPs in aging and neurodegenerative studies brings additional, sometimes unique information compared to other animal models.

## The primate brain advantage

In neuroscience, the global understanding of how brain works in physiological and pathophysiological conditions represents a major challenge. Brain evolution is characterized by a complex pattern of species similarities and differences. The neocortex, also called the “thinking” brain, represents the latest evolutionary stage and brings the capacity, for instance, of planning complex cognitive behaviors, personality expression, decision-making and moderating social behavior. These observations suggest a more complex circuitry that favors local connectivity (Herculano-Houzel et al., 2010) with cognitive consequences (Herculano-Houzel, 2012). Another important point is the important role played by the primate prefrontal cortex in “higher” brain functions, such as reasoning, judgment or social intelligence. Although the total prefrontal cortex volume is larger in humans than in other primates, suggesting that humans have more connections among prefrontal cortex neurons and consequently greater communication (Schoenemann et al., 2005), many of the genes expressed in human prefrontal cortex are also detected in the prefrontal cortex of NHPs (Marvanová et al., 2003). In rodents, the existence of the prefrontal cortex is still debated. However, it is clear that they lack functional areas involved in overall planning, such as the prefrontal granular cortex (Passingham and Wise, 2012). Moreover, rodents rely primarily on the use of their nose and whiskers for orientation, because their hearing and vision are much weaker than in NHPs. Conversely, NHPs are visually-oriented, like humans. They perceive the world first with their eyes, showing that their visual system is the main sense responsible for behavior. Spatial information is received through visual sensations (Maryanski and Turner, 1993). It is also possible to test NHP cognitive functions by using sophisticated go/no go procedures, such as automated cognitive test batteries, which are similar to those used in human studies on aging and Alzheimer's disease (Nagahara et al., 2010; Zürcher et al., 2010; Joly et al., 2014). These similarities between NHP and human brain in terms of anatomy, gene expression, neural circuitry, functional and cognitive abilities make of NHPs unique, valuable models for neuroscience research. They provide a more direct approach that can be translated to human diseases as they allow investigating higher intellectual functions with comparative endpoints (Sutcliffe and Hutcheson, 2012).

## Nonhuman primate models matter because they fill the gap between rodents and humans

Nonanimal experimental approaches play an important role in the identification/selection of candidate drugs; however, animal-based methods are required for toxicity testing. In addition, the safety of pharmaceuticals must be assessed using nonrodent models because these tests are carried out with the aim of protecting human patients in clinical trials. In Europe, all classes of pharmaceuticals must be tested in NHPs because NHP pharmacodynamic responses are close to those of humans. We will illustrate this point with four examples:

1. Developmental and reproductive toxicology effects are tested in NHPs (Chellman et al., 2009), most frequently in cynomolgus macaque (*Macaca fascicularis*) (Buse et al., 2003; Weinbauer et al., 2008) but also in rhesus macaques and marmosets, because of the similar pharmacological responses in NHPs and humans;
2. NHP retina has unique features (for instance, both NHPs and humans have a macula lutea/fovea) not found in other mammals (Stone and Johnston, 1981);
3. NHP blood coagulation system is more similar to that of humans than any other species (Abildgaard et al., 1971; Lewis, 1996);
4. NHPs are less susceptible to vomiting than other animal models (Weber, 2005). Therefore, in NHPs, vomiting will not decrease the exposure to the tested compound and will not confound the assessment of the early effects of a compound.

Another important phenomenon associated with drug testing in NHPs, although very difficult to deal with, is the inter-individual variability. This variability can be observed for instance in behavioral studies using the gray mouse lemur (Joly et al., 2006), in the response to treatments for Parkinson's disease in monkeys (Vezoli et al., 2011), and also in immune functions (Lebrec, 2013). NHP inter-individual variability poses real problems in terms of statistical evaluation of outcomes. However, it mimics what happens in humans and therefore helps us to better understand the variability within human populations.

## NHPs grow old like humans

NHPs have also significantly contributed to understanding aging and neurodegenerative diseases. Aging NHPs show striking similarities with elderly humans. Like in humans, age-related changes in the glutathione metabolic pathway have been observed in Old World simians (Rathbun and Holleschau, 1992) and in the gray mouse lemur, in which glutathione-synthase activity decreases and glutathione-peroxidase activity increases in the lens with age (reviewed in Languille et al., 2012). Aging gray mouse lemurs also present changes of the sensorial system, especially high susceptibility to cataract (the most frequent ocular lesions observed) (Beltran et al., 2007), alterations of the biological rhythms and of the endocrine system (Van Someren and Riemersma-Van Der Lek, 2007) and a progressive decrease of their motor capacities (Nemoz-Bertholet and Aujard, 2003). Interestingly, acceleration of seasonal rhythms (i.e., an annual cycle takes place in 8 months rather than 1 year by alternating long and short photoperiods) affects survival and longevity (Perret, 1997). This result suggests that longevity could be correlated with the succession of seasonal cycles rather than with a fixed biological age. This hypothesis is very interesting and it might apply also to human populations. Indeed, it has been reported that the accumulation of harsh winters may be responsible for a decrease in longevity (Robine et al., 2012).

Social interactions are fundamental in primates. For instance, Picq (1992) suggested that older gray mouse lemur females are less interested in social contacts than younger females. In rhesus monkeys, a decrease in social interactions with age has been observed (Heydecke et al., 1986). In chimpanzees, Goodall (1986) and Huffmann (1990) reported withdrawal from social interactions in older individuals. Sex-based differences in social interactions, social roles and social networks have also been observed in aging rhesus macaques (Corr, 2003). Altogether, these examples show that social interactions are of the utmost importance for aging studies in NHPs and should be used as clear readouts.

## The aging NHP and human brain transcriptomes are similar

Most of our understanding on the biological changes observed during aging comes from studies in rodents because they present clear advantages (short life span, fully characterized genetic aspects, easy genetic manipulation…). However, rodents and humans diverged much earlier than humans and NHPs, and this is likely to have led to fundamental differences in their aging processes (Messaoudi and Ingram, 2012). In a pioneering work, Loerch et al. (2008) compared the transcriptome of the cerebral cortex in aging mice, rhesus macaques and humans, providing a broad view of the evolution of aging mammalian brain. They found that only a small subset of age-related gene expression changes are conserved from mouse to human brain, whereas such changes are highly conserved in rhesus macaques and humans. Similarly, Marvanová et al. (2003) showed that more than 80% of genes detected in the human prefrontal cortex have similar expression profiles also in NHPs. Moreover, the study by Loerch et al. revealed that the major distinguishing feature is the dramatic, age-related increase in neuronal gene expression down-regulation, particularly of genes involved in neurotransmission, in humans compared to mice (Loerch et al., 2008). These major evolutionary changes in the primate cortex are potentially relevant when studying age-related changes in cognition and vulnerability to neurodegeneration. These observations have been recently corroborated by a transcriptomic analysis, using human DNA chips (Abdel Rassoul et al., 2010), of the gene expression profiles in the temporal cortex of young adults, healthy old animals and “Alzheimer's disease-like” (“AD-like”) gray mouse lemurs that naturally present the pathognomonic lesions of Alzheimer's disease. The temporal cortex was chosen because this region is connected to the hippocampus and to the frontal cortex, two structures that are critical for learning and memory and are altered in Alzheimer's disease. This study identified 47 genes that discriminated young from healthy old and “AD-like” animals. Functional categorization showed that most of the genes that were up-regulated in healthy old animals and down-regulated in “AD-like” animals belonged to metabolic pathways, particularly protein synthesis. These data suggest the existence of compensatory mechanisms during physiological brain aging that disappear in “AD-like” animals (Abdel Rassoul et al., 2010).

## Nonhuman primates naturally display Alzheimer's disease lesions such as amyloid plaques and aggregated hyperphosphorylated tau protein

All NHPs naturally display, to various extents depending on the species, the main pathognomonic lesions of Alzheimer's disease (AD): amyloid-beta (Aβ deposits, tau aggregation (reviewed in Heuer et al., 2012) and also cortical atrophy. For instance, Aβ plaques and/or tau aggregation have been found in rhesus monkeys (Uno and Walker, 1993; Walker, 1997; Bihaqi and Zawia, 2013), cynomolgus monkeys (Nakamura et al., 1998), great apes (Rosen et al., 2008), vervet monkeys (Kalinin et al., 2013), tamarins (Lemere et al., 2008) and gray mouse lemurs (Bons et al., 1995; Mestre-Frances et al., 2000). A N-terminal variant called pyroglutamate-3 Aβ, which is thought to play an early role in AD pathogenesis, has been detected in the brain of 13–32 year/old Caribbean vervets (Frost et al., 2013). In addition, based on neuron counting of the subcortical cholinergic basal forebrain regions (Smith et al., 1999) or imaging studies (Winkelmann et al., 2012), NHPs show cerebral atrophy and this feature has been linked to cognitive decline. For instance, in gray mouse lemurs, age-related decrease in spatial memory performance is related to atrophy of the hippocampus and entorhinal cortex (Picq et al., 2012), the two regions primarily affected in AD. Very recently, Darusman et al. (2014) found in aged cynomolgus monkeys a correlation between poor performances in memory tasks and hippocampus atrophy.

NHPs could also be useful to test new AD therapeutic approaches, for instance anti-amyloid beta (Aβ) immunotherapy. The idea is to combat AD by injecting the Aβ peptide, which is considered to play a central role in AD neuropathology, to trigger an immune response that eventually leads to the production of antibodies against Aβ. The first attempts in an AD transgenic mouse model showed that immunization with Aβ42 markedly reduced the AD-like pathology (Aβ plaque formation, neuritic dystrophy and astrogliosis) (Schenk et al., 1999). In a follow-up study in five aged Caribbean vervets, Lemere et al. (2004) validated this paradigm, showing a reduction of cerebral Aβ and gliosis. This major result generated a great deal of enthusiasm. However, administration of full-length Aβ self-antigen in humans (Elan/Wyeth AN-1792 trial) resulted in brain and spinal cord inflammation and possible micro-hemorrhages (Orgogozo et al., 2003; Gilman et al., 2005). As a consequence, less toxic Aβ derivatives have been developed in Tg2576 transgenic mice (Sigurdsson et al., 2001). We found that these Aβ derivatives elicit a substantial antibody response in gray mouse lemurs and, importantly, that this effect is reversible, thus enhancing the safety profile of this approach (Trouche et al., 2009). We have also shown iron accumulation in the choroid plexus with aging by MRI. This accumulation could be worsened by Aβ-immunization, and this observation should be a side effect that may be monitored in therapeutic trials (Joseph-Mathurin et al., 2013).

## Breakthroughs in prion research thanks to the use of NHPs

Prion diseases are fatal neurodegenerative diseases that affect humans and animals. Prion diseases are thought to be caused by transmissible pathogenic agents (“prions”) that can convert a normal cellular protein (called PrP^c^) into the pathogenic, transmissible form called PrP^Sc^ (reviewed in Fraser, 2014). Among the different forms of prion diseases, variant Creutzfeldt-Jakob Disease (vCJD) received considerable attention in the late 20th century because humans can be infected through oral consumption of animals infected by the bovine spongiform encephalopathy (BSE) prion strain. NHP-based studies have much contributed to advancing our knowledge on this disease (reviewed in Krasemann et al., 2012). For instance, the risk and the mechanisms associated with oral transmission have been studied using squirrel monkeys (Gibbs et al., 1980), cynomolgus macaques (Herzog et al., 2004; Lasmezas et al., 2005) and gray mouse lemurs (Mestre-Frances et al., 2012). These studies led to three major conclusions:

1. The asymptomatic incubation period may be extremely long (several years), much longer than in the case of intravenous or intracerebral infection;
2. The spectrum of tissues harboring BSE infectivity includes not only brain, but also the lymphoreticular system (spleen, lymph nodes, appendix, tonsils)
3. It is possible to estimate the food exposure risk.

These results highlighted the necessity of rapidly introducing health policy measures to prevent BSE transmission to humans. Furthermore, the use of NHPs not only improved our knowledge on the transmissibility of prion strains, but also allowed studying the fine molecular mechanisms involved. For instance, visual impairment, associated with retinal damage, is one of the first clinical signs of prion disease in *Microcebus murinus*. We have, therefore, developed an experimental model system based on the analysis of the retina of gray mouse lemurs to rapidly test prion-related neurotoxicity and to develop new therapeutic approaches (Torrent et al., 2010).

## The MPTP NHP models of neurodegeneration and the renaissance of Parkinson's disease research

Based on the observation of early-onset parkinsonism in users of a synthetic opioid, 1-methyl-4-phenyl-1,2,3,6-tetrahydro-pyridine (MPTP), a compound with similar neurotoxic properties, was further developed. Studies in squirrel monkeys, rhesus macaques and marmosets showed that MPTP injections leads to dopamine depletion in the *substantia nigra pars compacta* followed by drastic cell loss, resting tremor, rigidity and bradykinesia, like in humans with Parkinson's disease (reviewed in Fox and Brotchie, 2010). Conversely, rodents are not sensitive to MPTP (Capitanio and Emborg, 2008) and MPTP injections do not lead to the full spectrum of Parkinson's symptoms in these animals. Importantly, the MPTP NHP models allowed the identification of the neural circuits affected in Parkinson's disease, specifically the role of excessive activity in the subthalamic nuclei (Bergman et al., 1990), leading to the development of ablative procedures (Baron et al., 2002) and of deep-brain stimulation of these nuclei (DeLong and Benabid, 2014). This latter experiments would not have been developed without knowing the physiology of the basal ganglia in nonhuman primates (Baron et al., 2002). The MPTP NHP models are also used to discover neuroprotective compounds for Parkinson's and other diseases associated with motor deficits, such as Huntington disease or Amyotrophic Lateral Sclerosis (Philippens et al., 2010; Uchida et al., 2012).

## Conclusion and perspectives

Due to their genetic proximity to humans and their highly developed social skills, NHPs are extremely valuable as experimental animal models. However, as the number of available animals is restricted for ethical reasons and also because of the high cost and large space required for breeding colonies, NHPs should only be used when no other suitable method is available to fill the gap of our knowledge. Developing the use of small and relatively short-lived NHPs, as proposed by Austad and Fischer (2011), could lower the costs per animal and facilitate the colony management and growth. They could be used in preclinical studies with clear reachable endpoints or in long-term follow-up studies (equivalent to phase IV in clinical trials) (Lemere et al., 2004; Trouche et al., 2009). NHP unique contribution to aging and neuroscience research is well exemplified (Capitanio and Emborg, 2008; Austad and Fischer, 2011; Bihaqi and Zawia, 2013); however, they are also extremely valuable for studying other diseases, such as spinal cord injury (Courtine et al., 2007), infectious diseases (reviewed in Gardner and Luciw, 2008), respiratory diseases (Curths et al., 2014), and also for pharmacological studies (Nader and Banks, 2014) because their pharmacodynamic and pharmacokinetic parameters are closely related to those of humans.

In any case, rodent (or other small animal models) and primate experimental models need to be used in parallel in order to obtain robust and complementary information. Alongside other models, nonhuman primates should have a unique place in the overall aging and neurodegenerative research strategy.

### Conflict of interest statement

The authors declare that the research was conducted in the absence of any commercial or financial relationships that could be construed as a potential conflict of interest.
